# The Royal Hospital for Incurables, Putney

**Published:** 1892-07-30

**Authors:** 


### THE ROYAL HOSPITAL FOR INCURABLES, PUTNEY.

In view of the special meeting held on the 22nd inst., referred to elsewhere, we think it may be useful to reproduce the following summary of the evidence, from pp. 57 to 59 of the Lords' Committee's Report:—

Royal Hospital for Incurables, Putney.

This hospital was founded, 1854, by public subscription. The secretary, who has filled that position from the commencement, receives a salary of £500, without board or lodging, and does not reside in the hospital. There are 218 inmates—38 men, 180 women. Pensions of £20 per annum are allowed to poor people in any part of the country, to the amount of £11,000, the pensioners being elected from the list of applicants.

The management is as follows. There is an annual meeting of governors (who are qualified by half-guinea subscription per annum, or a single donation of £5 5s.); all governors may attend. There is no quarterly meeting, but half-yearly meetings are held for the election of candidates for indoor and outdoor relief. There is a board of management consisting of 20 governors, with a quorum of five; from this board is appointed a house committee. The board sit once a fortnight; the house committee once a week; six or seven governors usually attend. The business is to take cognizance of all principal matters in connection with the institution; they interview and receive reports from the matron, who is the principal officer, and the steward.

Individual members of the committee occasionally visit the dinners; these visits are said to be so occasional that an average could not be given offhand, but (the secretary thought) quite twice a year. As to the suggestion that these visits should be twice a-week, he thought "It would not be reasonable, because that would be calling gentlemen from their homes or from London to do that which they would not have time to do."

Books are laid before the committee and seen and signed; they do not go over each item, there would not be time. Other duties of the committee are to hear reports from the medical officer and from the seaside home, and requests for leave, and to examine the staff gate-book. The reports are in writing, and are read to the committee. There is no visiting committee, but governors living in the neighbourhood do visit; no written report is made by such visitors; though there are no fixed visiting governors, the institution, the secretary said, was always open to the public and the governors, the house was freely open to everybody.

Mr. Burdett, on this point, said that this was the only institution he had ever had any trouble in getting permission to enter; every impediment was placed in his way in ascertaining on what principle the institution was managed internally. For some time he was refused a plan, but he ultimately received one. Permission was denied to himself, his architect, or his secretary, to enter the building. His experience in regard to this institution was unique. Even in Russia they gave him greater facilities for entering a hospital than he could get from the Royal Hospital for Incurables at Putney.

The following extracts are taken from the secretary's evidence. A patient may write a complaint to the chairman; a complaint may be put in the matron's book of requests; a patient may desire a visit from one of the committee. The matron is German by birth, and has a salary of £200 with board and lodging; she was trained on the Nightingale system, and was, at one time, in Sir P. Dunn's Hospital in Dublin. No advertisement of the vacancy was made at the time of her appointment; she was introduced by one of the members of the committee, having been a governess in his family; it would be libellous to say she had a terrible temper; but she can exhibit temper. A complaint was made two or three years ago of her speaking violently to a patient, and she pleaded an extremely irritating cause. "The matron is supreme in the absence of any of the committee and the secretary, but the secretary does not claim to have authority in the house." That is her province. The matron, however, "of course applies to the secretary for advice, and he takes cognizance of everything and anything that goes on." Complaints by a patient with regard to the nursing, or the matron, would be made to the matron. It is her duty to remedy anything within her own judgment and power. The matron reports to the committee all changes that take place, and the reason of them; she selects, engages, and dismisses the nurses, and reports to the committee; it practically did not happen that the nurses appealed from the matron to the committee; any offence would be dealt with off hand by the matron. The matron is, principally, responsible for the ventilation of the wards.

There is no nursing committee. There are two grades of nurses; those of the first grade, in all five of them, are trained nurses, three on duty by day and one by night. In the second grade the nurses are untrained, but have some notion of nursing; they are regarded as attendants on the patients. The witness did not consider more trained nurses to be required. There is only one trained nurse in charge of one corridor of 40 beds by day; by night, one night nurse and two assistant nurses for the whole female side.

No nurses defaulters' book is kept. The nursing is entirely under the matron.

There are male attendants, mostly old soldiers, who have been employed in lunatic asylums; they are mostly employed in lifting patients.

The number of women is 180 to 38 men. There is no committee of female governors; the suggestion had been made, but the witness considered that some of their patients were "a little injured by over sympathy;" he thought a ladies' committee would be disastrous, and a "thoroughly competent matron, a skilled woman, such as they had now, would probably not at all please a ladies' committee."

A matron would probably not submit to the supervision of a committee of ladies. The witness admitted that a committee of ladies would probably discover much about the matron, and the management, of which he was now ignorant.

The steward receives £150, and board and lodging, he takes in the provisions and issues them, and has control of the male servants.

Food is contracted for; the contracts are made on the recommendation of the Finance Committee; the tender is not open, but a select number of tradesmen are sent to, and the witness considered that experience was against issuing tenders broadcast. The meat contract had been for some years in the hands of one man, and previously the contract was given alternately to him and another man. He had compared the prices paid with other institutions, "but not very frequently, because we are our own judges in the matter, and we have every reason to believe, at least we have good reason to believe, that the tenders are genuinely put in."

The drains were under the steward's supervision; he was not a sanitary engineer, but if necessary, would consult the architect, who would know as much as any architect about drains.

The medical officer receives £200 per annum, and is non-resident; a resident medical officer would not find sufficient to do. His reports are not filed, but he keeps the history and treatment of every case. He takes outside practice; if engaged when wanted, his partner would come, but this seldom occurs.

There is a consulting staff. There is no paid chaplain, but voluntary service is performed from outside; the witness thought that a paid chaplain would lead to denominational difficulties.

In 1890 a letter was addressed by the Duke of Portland to the Board, bringing to their notice the "very general complaints which he heard on all sides about the management" of this institution. The points referred to in the letter are "food," "want of supervision," "management," and "general." Under the last head came "time for patients' meals," "neglect of religious needs of inmates." The points were replied to seriatim by the secretary, on behalf of the treasurer and the board, and no further communication being received from the Duke of Portland, the management considered that the replies were satisfactory. One striking discrepancy between the reply of the managers and the evidence given by Mr. Andrew before the committee is, that whereas the managers stated the meat contracts were open to competition, Mr. Andrew stated that such was not the case. In regard to the complaints and the reply, Mr. Andrew's evidence was that investigation was made. The committee of management went into the matter, as the house committee, on the spot. The evidence of the matron was taken, but the witness could not charge his memory as to others. He did not think it a case where evidence was necessary, and considered that the complaints were fully and fairly dealt with.

The receipts for the past year were £44,509; expenditure (including pensions, £11,129) about £28,000; balance, £16,509.

### Conclusion of Lords Committee

It will be remembered that the committee arrived at the following conclusion in regard to this hospital:—

The committee think it their duty to invite particular attention to the case of the Royal Hospital for Incurables at Putney. While in receipt of very large support, having a surplus in 1889 of £16,000, the authorities of this hospital appear to be incapable of effecting reforms, and are extremely resentful of external observation. In regard to this hospital the committee would strongly recommend reforms in this direction: That a resident medical officer should be appointed with general control in absence of the committee, as is the case in the poor law infirmaries; that a ladies' committee should be appointed, as a large majority of the patients are females; that all nurses should be hospital-trained; that the contracts for food, and stores of all kinds, should be by open tender, and that the general supervision by the committee of governors should be greatly increased. The objects of this charity are excellent, but until the management is thoroughly reformed, the committee regret that they feel bound to add that the institution is not one which can be commended.

